# Reconstruction after resection of C2 vertebral tumors: A comparative study of 3D-printed vertebral body versus titanium mesh

**DOI:** 10.3389/fonc.2022.1065303

**Published:** 2022-12-19

**Authors:** Panpan Hu, Suiyong Du, Feng Wei, Shuheng Zhai, Hua Zhou, Xiaoguang Liu, Zhongjun Liu

**Affiliations:** ^1^ Department of Orthopedics and Beijing Key Laboratory of Spinal Disease Research, Peking University Third Hospital, Beijing, China; ^2^ Department of Spine Surgery, 521 Hospital of Norinco Group, Xi’an, China

**Keywords:** primary spine tumor, C2 vertebra, total resection, 3D printing, titanium mesh, artificial vertebral body

## Abstract

**Background:**

Surgical resection of C2 vertebral tumors is challenging owing to the complex anatomy of C2 vertebrae and the challenges to surgical exposure. Various surgical approaches are available, but some are associated with excessively high risks of complications. An additional challenge is reconstruction of the upper cervical spine following surgery. In the last decade, additive-manufacturing personalized artificial vertebral bodies (AVBs) have been introduced for the repair of large, irregular bony defects; however, their use and efficacy in upper cervical surgery have not been well addressed. Therefore, in this study, we compared instrumented fixation status between patients who underwent conventional titanium mesh reconstruction and those who underwent the same resection but with personalized AVBs.

**Methods:**

We performed a retrospective comparative study and recruited a single-institution cohort of patients with C2 vertebral tumors. Clinical data and imaging findings were reviewed. Through data processing and comparative analysis, we described and discussed the feasibility and safety of surgical resection and the outcomes of hardware implants. The primary outcome of this study was instrumented fixation status.

**Results:**

The 31 recruited patients were divided into two groups. There were 13 patients in group A who underwent conventional titanium mesh reconstruction and 18 group B patients who underwent personalized AVBs. All patients underwent staged posterior and anterior surgical procedures. In the cohort, 9.7% achieved total en bloc resection of the tumor, while gross total resection was achieved in the remaining 90.3%. The perioperative complication and mortality rates were 45.2% and 6.5%, respectively. The occurrence of perioperative complications was related to the choice of anterior approach (*p* < 0.05). Group A had a higher complication rate than group B (*p* < 0.05). Four patients (4/13, 30.8%) developed hardware problems during the follow-up period; however, this rate was marginally higher than that of group B (1/18, 5.6%).

**Conclusions:**

Total resection of C2 vertebral tumors was associated with a high risk of perioperative complications. The staged posterior and retropharyngeal approaches are better surgical strategies for C2 tumors. Personalized AVBs can provide a reliable reconstruction outcome, yet minor pitfalls remain that call for further modification.

## 1 Introduction

The C2 vertebral body is one of the most common sites of primary spinal tumors; yet, this area has complicated anatomic conditions, with the existence of large arteries, excessive venous plexi, and important neurological structures (1). Technically, it is difficult to perform total en bloc resection (TER) of tumors in the C2 vertebral body. To achieve this surgical goal, surgeons generally choose between a combined or staged anterior and posterior approach to fully expose the lesion (2–5). Gokaslan and colleagues described the procedure of a single posterior TER of C2 vertebral tumors, with the two cases both receiving satisfactory outcomes (6, 7). Some additional authors have also described techniques of the single anterior approach (transoral, transmandibular, or retropharyngeal) for C2 vertebral tumors, whereas most of the cases barely achieved intralesional or gross total resection (GTR) (8–11).

Regardless of the surgical approach, the TER of C2 vertebral tumors is technically demanding and accompanied by a high risk of severe complications including cerebrospinal fluid leakage, vascular ruptures, paralysis of the diaphragm, respiratory dysfunction, ventilator dependence, wound problems such as an unhealed pharyngeal wall, and neurological deficits (3, 4, 7, 12–14). Moreover, hardware problems and even failures have been shown to be excessively high. Wei et al. (2016) reported that nearly 50% of their cases involving TER of C2 vertebral tumors had problems of bony malunion and disunion, and one-third of the cases had fixation failure (4). In an impressive case report by Rhines and colleagues (2005), the patient developed apparent migration of the graft and plate during the hospital stay; thus, an emergent revision was arranged (12). Singh et al. (2020) have also described their experience with the technique of modified titanium mesh and iliac crest graft, with solid fusion achieved after 18 months of follow-up (15). In 2016, Xu et al. introduced a customized 3D-printed artificial vertebral body (AVB) to reconstruct the upper cervical spine after the total resection of C2 Ewing sarcoma (16). Personalized 3D-printed AVBs have since then been widely utilized in column reconstruction after the resection of spinal tumors (10, 17–22). However, considering that few centers can perform TER of C2 tumors and/or have access to 3D-printed AVBs, there is a lack of specific comparative studies, and the efficacy and superiority of personalized AVBs have therefore not been fully addressed. Thus, in this study, we conducted a comparative analysis between 3D-printed AVBs and conventional titanium constructs.

## 2 Materials and methods

### 2.1 Patient inclusion

This was a retrospective comparative study. Patients with C2 vertebral tumors were reviewed from our institutional database of spinal tumors, and all patients were screened for eligibility. The inclusion criteria were as follows: (1) undergoing GTR or TER, (2) receiving anterior column reconstruction using either titanium meshes or AVBs, and (3) being regularly followed up until death or beyond 12 months. This study recruited 31 consecutively treated patients between January 2009 and December 2020. According to the methods of anterior reconstruction, two groups were allocated: group A, titanium mesh; and group B, personalized AVBs. Clinical records and imaging data of the recruited patients were reviewed and analyzed. The study was approved and supervised by our institutional ethics committee and all participants provided informed consent.

### 2.2 Preoperative evaluation and preparation

The routine preoperative imaging set included plain radiography, computed tomography (CT), CT angiography (CTA), magnetic resonance imaging (MRI), and positron emission tomography- CT. CTA was necessary to determine the position of the vertebral arteries (VA). In this case series, CT-guided biopsy was performed for pathological diagnosis. For patients with a large tumor mass or potential involvement of the VA, preoperative embolization of tumor lesions and feeding vessels was performed to reduce intraoperative blood loss.

The preparation of personalized AVBs has been reported previously (22). After acquiring the patients’ 1-mm-thin layer CT scans, the data were imported into MIMICS software (version 15.0; Materialize, Leuven, Belgium) for prosthesis design. This procedure was performed under the supervision of senior surgeons. The porous prosthesis was fabricated from Ti6Al4V powder by electron beam melting (Arcam EBM System; Arcam, Mölndal, Sweden). The diameters of the pores and wires were set at 600 ± 200 μm and 550 ± 200 μm, respectively, and the average porosity rate was 50%–80%.

### 2.3 Surgical procedures

All patients underwent staged posterior and anterior surgeries to achieve GTR or TER goals. The surgery was performed by our senior authors, namely, the FW and ZL teams. During the posterior procedure, the most important goal was to isolate the neurological structures and the VA. First, we resected the C2 lamina and lateral masses and exposed the spinal cord and bilateral nerve roots. Subsequently, the C2 transverse foramen was gently palpated using nerve probes, and its posterior and lateral walls were carefully removed in a piecemeal manner. Generally, a 1-mm Kerrison rongeur is first used to open a fissure, and then a high-speed drill or ultrasonic bone scalpel can be employed under the tight protection of the VA. After completing this step, we were able to remove the bilateral pedicles from the vertebral body. In some cases, it was necessary to ligate one side of the VA during the surgery to achieve TER of the tumor lesion. Posterior fixation was accomplished using an occipital or C1–C4/C5 screw-rod system.

**Figure 1:**
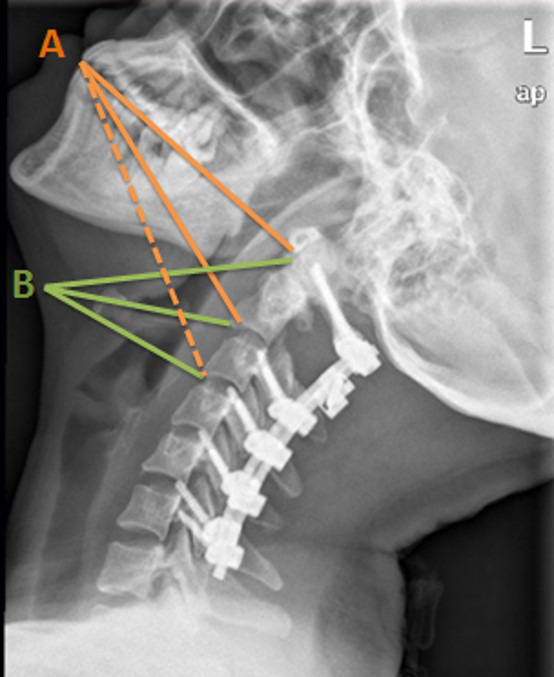
Illustration of longitudinal exposure range. Orange lines **(A)** represent the transoral approach: solid lines indicate areas that this approach was able to reach and the dotted line refers to the areas out of reach. Green lines **(B)** indicate where this approach can reach.

The anterior procedure is typically completed through the high retropharyngeal and transoral approaches (**Figure 1**). However, in case #A8, we used an aggressive transmandibular approach to achieve intralesional resection of the C2 chordoma (**Supplementary Table**). During the anterior procedures, we predominantly performed extracapsular dissection to avoid minimal residual tumors. First, we transected the bilateral musculus longus coli to expose the transverse process and carefully probed the transverse foramen. Then, we resected the anterior and lateral walls and isolated the VA using a Kerrison rongeur with or without powered tools. Generally, we transected the odontoid process at the cranial end, although this may have constituted intralesional manipulation in some cases. At the caudal end, we removed the C2/3 or C3/4 intervertebral discs to preserve an intact tumor margin. At this time, the entire vertebral body was released and removed as an entire mass.

### 2.4 Anterior column reconstruction

**Figure 2:**
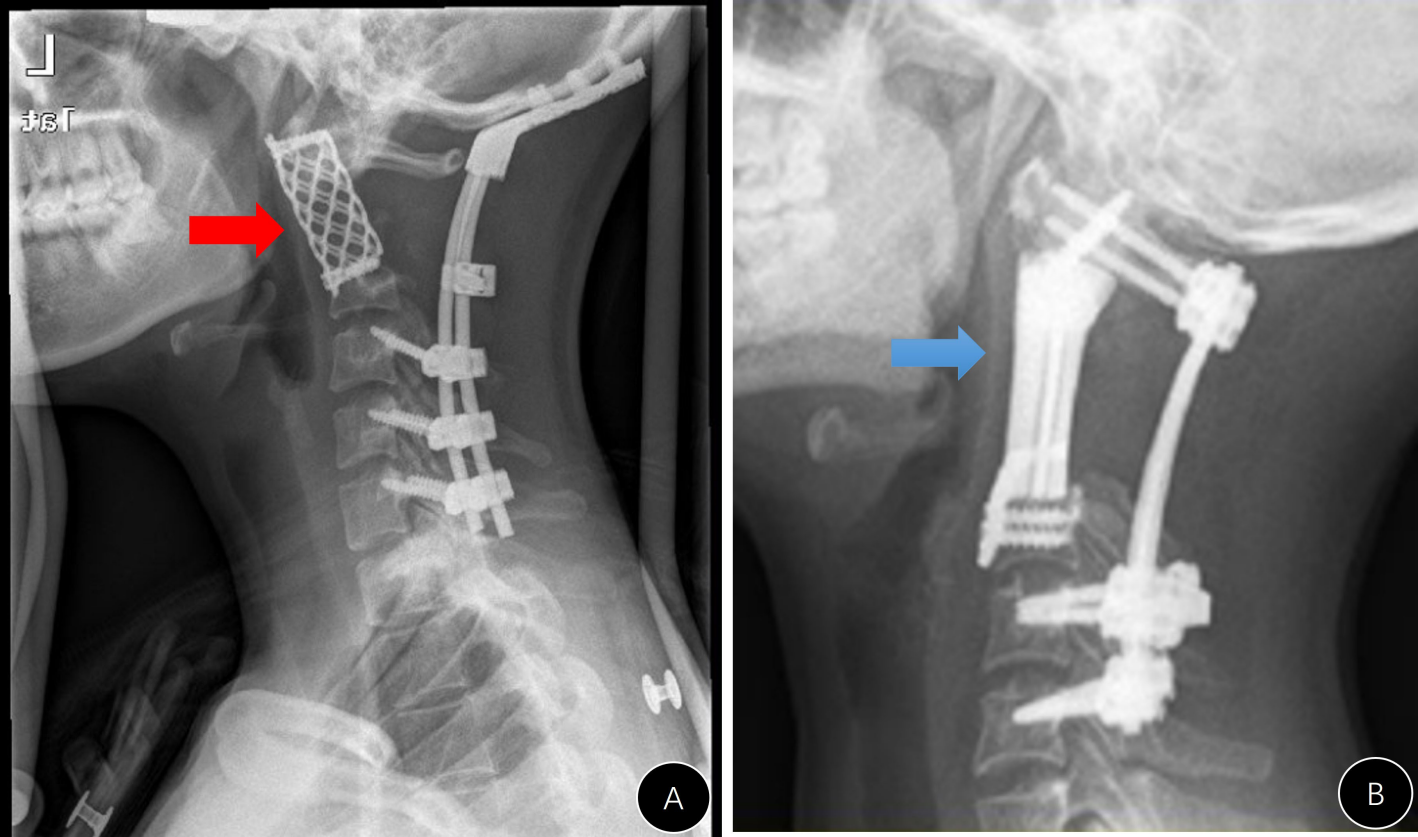
Two different anterior reconstruction materials. **(A)** Stand-alone titanium mesh; **(B)** 3D-printing artificial vertebral body.

In group A, we used cylindrical titanium mesh with a plate or stand-alone modified mesh (**Figure 2A**). In group B, patient-tailored AVBs were fabricated by 3D printing (**Figure 2B**). The choice of anterior reconstruction material was not randomized. Customized AVBs have been used in most cases since 2015, before which titanium mesh had been used exclusively.

### 2.5 Follow-up and data collection

The patients were regularly followed up at our clinic, with visit windows of 3, 6, and 12 months after the operation and lifelong assessments conducted annually. At each visit, we evaluated symptomatic improvement and performed imaging examinations including radiography, CT, and MRI. Positron emission tomography-CT was only indicated when evidence of tumor relapse emerged.

This study used instrumented fixation as the primary outcome indicator. Other data collected included those regarding demographics, surgical details, complications, tumor pathologies, staging [using the Enneking and Weinstein–Boriani–Biagini systems (23) systems], and patient survival status.

### 2.6 Statistical analysis

Data analysis was performed using IBM SPSS Statistics for Windows Version 20 (IBM Corp., Armonk, NY, USA). Data were presented as percentage, mean ± standard deviation, or median (range). The Student’s *t*-test and Pearson’s *χ* ^2^ test (or Fisher’s exact test) were used to compare different groups. Statistical significance was set at *p* < 0.05.

## 3 Results

### 3.1 Demographics and pathologies

The detailed data for each case are presented in the **Supplementary Table**. There were 13 cases in group A and 18 in group B. The average patient age was 43.3 years in group A and 38.2 years in group B (**Table 1**). Neck pain was the most common clinical complaint, and other symptoms included neurological deficits (six cases), torticollis (two cases), dysphagia, and dyspnea. Pathologically, this cohort included 14 cases of chordoma, 11 cases of giant cell tumor, 2 cases of osteoblastoma, and 1 case each of paraganglioma, Ewing sarcoma, hemangiopericytoma, and Schwannoma. Two patients died after the postoperative hospital stay, and the other patients were followed up beyond 12 months.

**Table 1:** Summary of data and comparison between the two groups.

Items		Group A (*n* = 13)	Group B (*n* = 18)	*p*-values
Age (years)		43.3 ± 3.7	38.2 ± 3.8	0.356
Pathologies (*n*)		Chordoma: 6	Chordoma: 8	
		GCT: 3	GCT: 8	
		OB: 2	PGL: 1 case	
		HPC: 1	ES: 1	
		Schwannoma: 1		
Surgical approaches				
	Posterior	mid-line: 13	mid-line: 18	
	Anterior	RP: 3	RP: 17	
		TO: 7	TO: 1	
		TM: 1		
		TO/RP: 1		
		TO/TC: 1		
Fixation and reconstruction				
	Posterior	Screw-rod: 13	Screw-rod: 18	
	Anterior	Mesh alone: 8	AVB: 18	
		Mesh/LP: 5		
Bleeding volume (ml)				
	Posterior	896 ± 150	625 ± 80	0.098
	Anterior	1,384 ± 232	603 ± 132	0.008*
Operative time (min)				
	Posterior	265.9 ± 68.7	239.8 ± 34.6	0.325
	Anterior	269.9 ± 91.6	222.1 ± 37.0	0.138
Tumor margins (*n*)				
	Intralesional	13	15	
	Marginal	0	3	
Halo vest (*n*)		10	3	0.003*
Complicated events (*n*)		9	5	0.022*
Hardware problems (*n*)		4	1	0.060

*Significantly different at p < 0.05.

GCT, giant cell tumor; OB, osteoblastoma; PGL, paraganglioma; HPC, hemangiopericytoma; ES, Ewing sarcoma; RP, retropharyngeal; TO, transoral; TM, transmandibular; TC, transcervical; AVB, artificial vertebral body; LP, locking plate.

### 3.2 Surgery-related data

**Figure 3:**
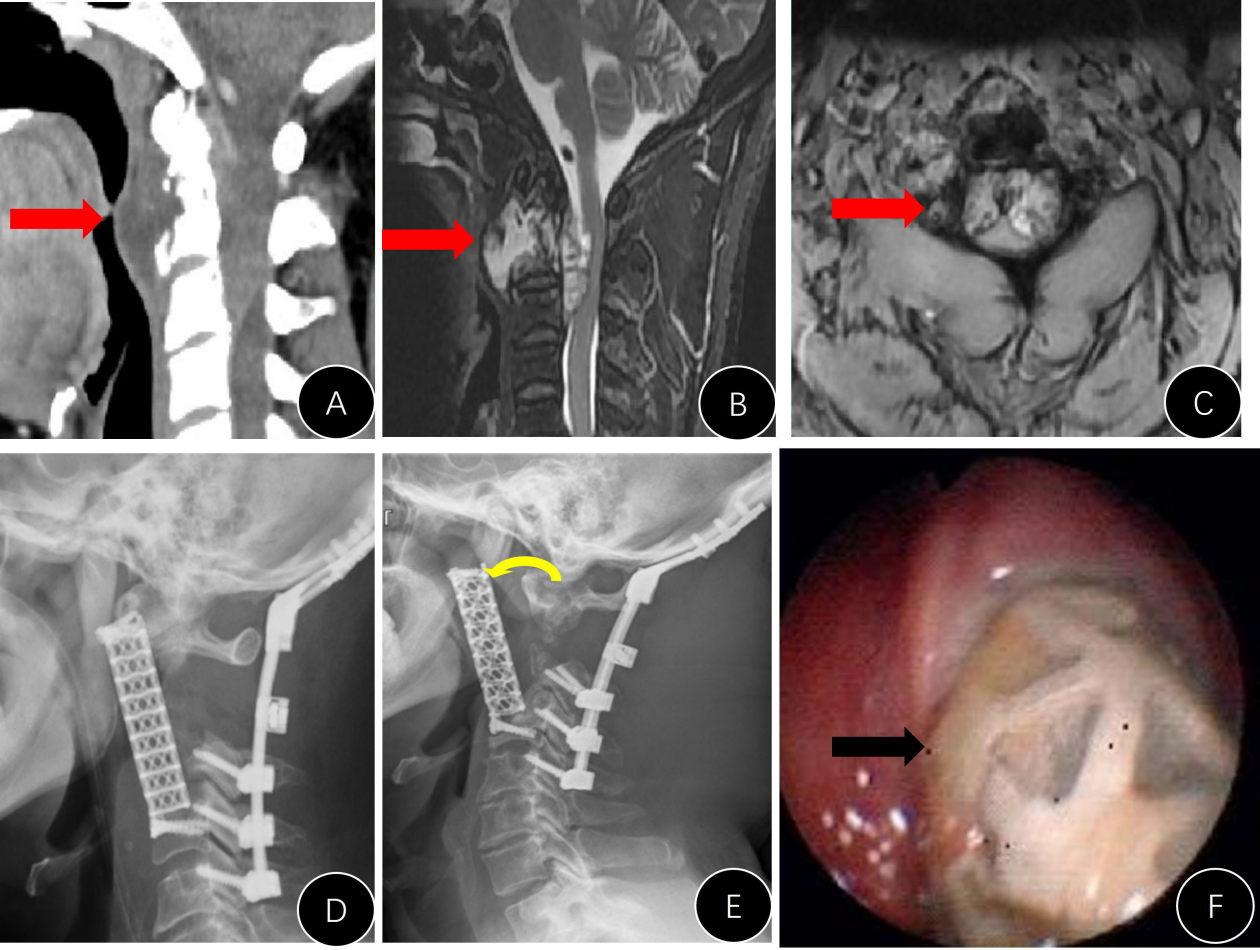
Presentation of #A9. **(A–C)** Preoperative CT and MRI images; **(D)** postoperative lateral x-ray; **(E)** lateral x-ray 3 years after the operation (anterior rotation of the titanium mesh); **(F)** unhealing of the pharyngeal wall with mesh exposure.

In group A, all patients underwent midline incision, and anterior procedures included retropharyngeal (three cases), transoral (seven cases), transmandibular (one case), and combined approaches (two cases) (**Table 1**). All patients who underwent posterior procedures received screw-rod fixation. The anterior reconstruction materials included a modified titanium mesh in eight cases (**Figure 3**) and a mesh plus locking plate in five. Additionally, we sacrificed the unilateral nerve root in five patients and ligated one side of the VA in three because of tumor invasion. Specifically, six patients (46.2%) underwent tracheotomy during the operation for better respiratory management, and one underwent preoperative vascular embolization to reduce intraoperative blood loss (case #A4). After the operation, 10 patients (76.9%) wore a halo vest (the majority for a minimum of 3 months) to consolidate the internal fixation.

**Figure 4:**
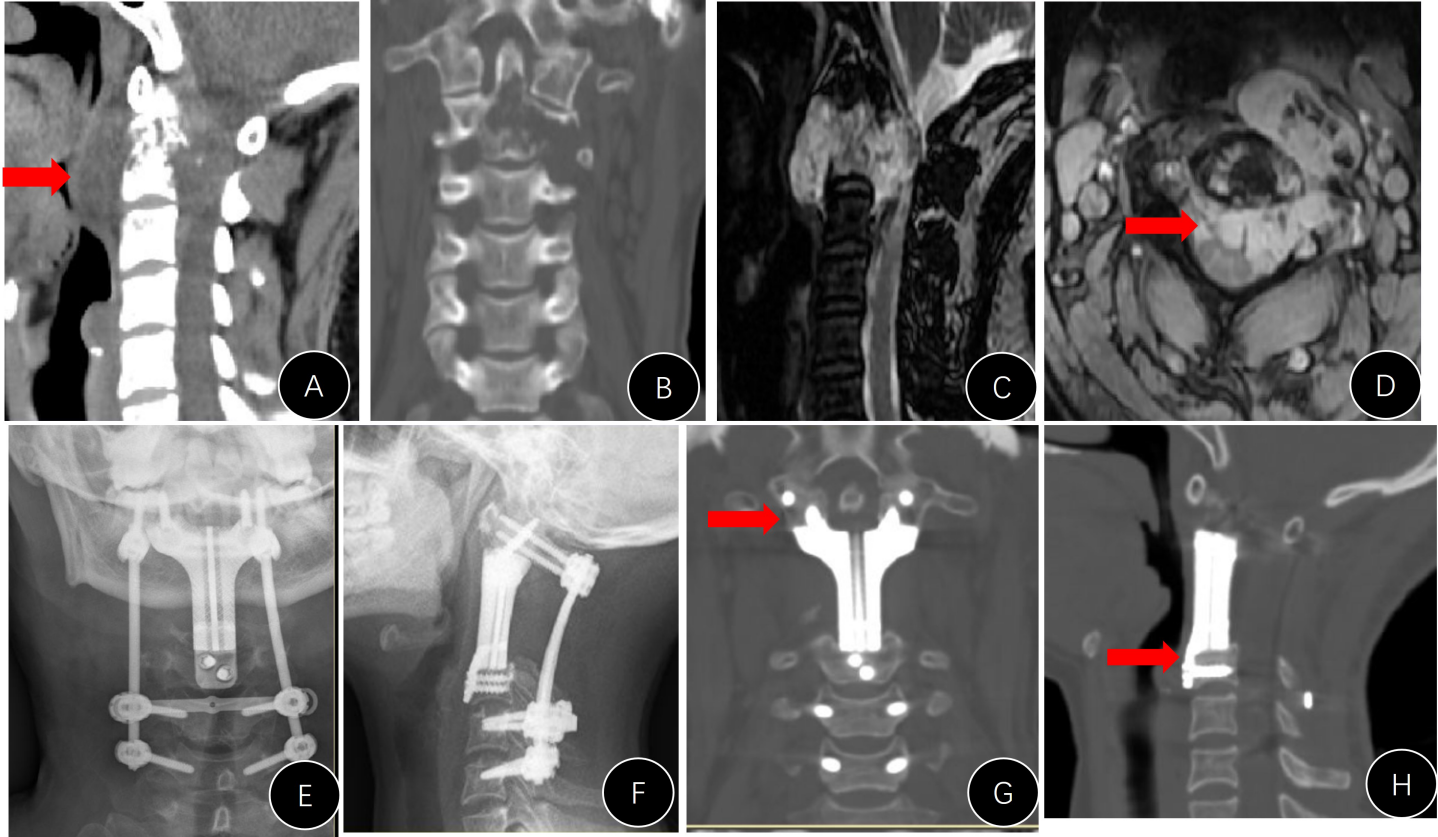
Presentation of case #B15. **(A–D)** Preoperative CT and MRI images; **(E, F)** postoperative x-rays; **(G, H)** CT reconstruction films 1 year later, indicating that the implant was well attached to the C1 lateral mass and superior endplate of C3.

In group B, 17 patients (94.4%) underwent the retropharyngeal approach and 1 (#B4) underwent the transoral approach during the anterior procedures. Personalized AVBs were used as exclusive column constructs (**Figure 4**). During the operation, we ligated one side of the VA (case #B5) because of an inadvertent injury. One patient underwent preoperative vascular embolization to reduce intraoperative blood loss (case #B7). After the operation, only three patients (16.7%) wore the halo vest for 3– 8 weeks, which was a lower number than in group A (*p* < 0.05).

### 3.3 Postoperative and follow-up events

Perioperative complications developed in 14/31 patients (45.2%) (**Table 1**). The major complications included cerebrospinal fluid leakage (5/31 cases, 16.1%), wound problems (8/31 cases, 25.8%), cardiac events (case #A8), and vascular events (case #A11 and #B12). Three patients (3/18, 16.7%) in group B had respiratory dysfunction after the operation and required a temporary tracheotomy, one of whom died of respiratory failure and septic shock (case #B16). In group A, one patient (case #A11) died of postoperative hemorrhagic shock. This patient showed consistent drainage of fresh blood and signs of circulatory dysfunction; emergency exploratory surgery was performed, yet an active bleeding spot could not be detected. Thus, the patient unfortunately died of hemorrhagic shock. The overall mortality rate in the cohort was 6.5% (2/31).

Patients in group A had a higher incidence of perioperative complications than those in group B (*p* < 0.05, **Table 1**), and patients who underwent retropharyngeal approaches (30.0%, 6/20) had a lower incidence of perioperative complications than those who underwent transoral and transmandibular approaches (72.7%, 8/11) (*p* = 0.022).

### 3.4 Internal fixation outcomes

**Figure 5:**
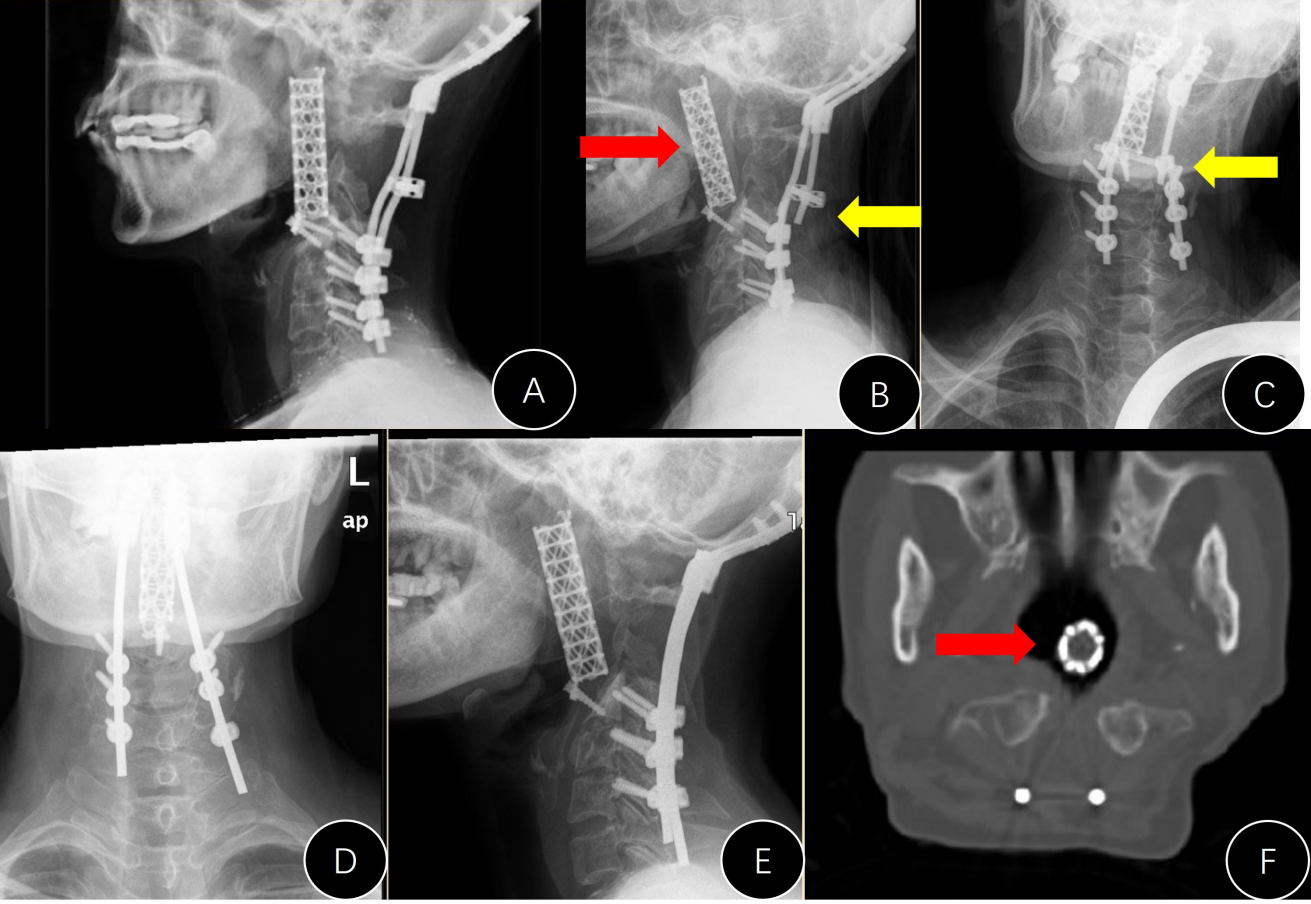
Presentation of case #A6. **(A)** Postoperative lateral x-ray; **(B, C)** x-rays 7 years later (anterior movement of titanium mesh and the breaking of both rods); **(D, E)** revision surgery to replace the rods; **(F)** mesh shifted into the pharyngeal cavity.

During follow-up, there were four cases (4/13, 30.8%) in group A with emerging internal instrument-related complications (**Table 1**). This ratio was marginally higher than that in group B (*p* = 0.060, **Table 1**). The stand-alone titanium mesh in case #A1 was observed to be malpositioned during the follow-up, and the modified meshes in cases #A3 and #A9 (**Figure 3E**) did not have a solid anchor at the cranial end and moved forward. Additionally, the titanium mesh in case #A6 did not fuse at all and completely loosened, and the posterior rods became broken. The broken rods were replaced during the revision operation, but the mesh was left untouched (**Figure 5**).

**Figure 6:**
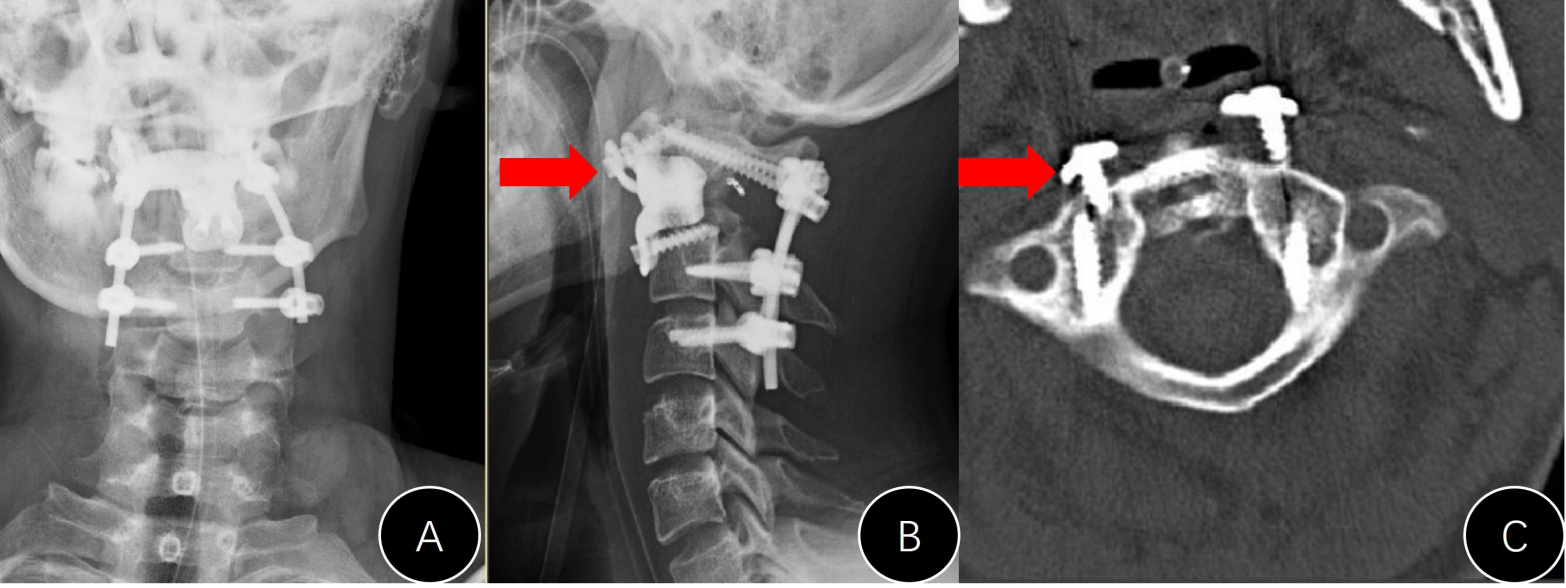
Presentation of case #B7. **(A, B)** Postoperative x-rays (unsatisfactory cranial attachment to C1 anterior arch); **(C)** CT film revealed that the cranial screws were not completely nailed in.

Only one case in group B presented with an instrument problem (case #B7). We found it difficult to nail the C1 screws during the operation and had to leave them malpositioned (**Figure 6**). However, the AVB in this patient did not loosen or move during follow-up.

## 4 Discussion

To the best of our knowledge, this report contains one of the largest single-center cohorts of C2 vertebral primary tumors. In addition, this article provides a comparative study between 3D-printed AVBs and conventional titanium constructs and demonstrates the efficacy and merits of AVBs with more solid evidence.

However, total resection of C2 vertebral tumors was associated with a high risk of perioperative complications. Staged posterior and retropharyngeal approaches were shown to be better surgical strategies for C2 tumors. Personalized AVBs can provide a reliable reconstruction outcome, yet minor pitfalls remain.

### 4.1 Surgical challenges and risks

Conventional radiotherapy is usually ineffective as a primary or adjuvant therapy after intralesional resection of malignant or aggressive tumors such as chordomas and GCT (24). However, more evidence has shown that modern radiotherapy techniques, such as stereotactic radiotherapy, provide durable local control as adjuvant therapy or even as the primary treatment for cases that are unable to undergo surgical resection (25). Theoretically, TER is the principal surgical goal for aggressive primary spinal tumors (14, 26, 27). A study based on the AOSpine Knowledge Forum Tumor database (28) revealed that an Enneking-appropriate (EA) surgical strategy for chordoma can significantly decrease locoregional recurrence and prolong the overall survival of patients. Although we attempted EA surgeries in all recruited patients, only three cases (9.7%, 3/31) achieved marginal TER. According to Zhong et al. (2021), TER can be achieved only for tumors localized within the vertebral body or odontoid process (29).

Owing to the complex anatomic structure of C2 and the existence of VA, resection surgery for C2 vertebral tumors is technically demanding and has an excessively high risk of complications (2–4, 7, 12–14). In this series, nearly half of the patients developed moderate-to-severe perioperative complications. This ratio is much higher than that for tumor resection surgery in other spinal segments (14, 20, 22). In our study, all patients underwent sequential posterior and anterior approaches to acquire more space for surgical manipulation and direct visual supervision. In previous studies, two-stage surgeries had a higher total bleeding volume than single-staged surgeries (6, 7, 9, 10). However, this strategy is still a better choice for most C2 vertebral tumors as it spares internal time for physical recovery between the two procedures.

Injury to the VA is one of the most severe complications in the resection of C2 vertebral tumors. In this study, the risk of inadvertent VA injury and ligation was high, and one case died of massive blood loss. Preoperative VA embolization can reduce operative bleeding, but it is difficult to perform in the upper cervical area (30, 31). According to Johns Hopkins (2020), the sacrifice of VA is only chosen in cases of complete encasement of the artery (32). Therefore, there are two important concerns regarding VA sacrifice: (1) it is technically possible to perform TER of C2 vertebral tumors and (2) VA is completely encased by the tumor. Otherwise, we should avoid arbitrarily sacrificing the VA.

### 4.2 Surgical approaches matter and retropharyngeal approach is safe

Previous studies have introduced a variety of surgical approaches for the upper cervical spine such as posterior, lateral/far-lateral, retropharyngeal, bilateral transcervical, transoral, endoscopic endonasal, and circumglossal approaches (2–4, 8–13, 33–36). In our center, we choose a posteroanterior approach as it provides a better visual field and simplifies surgical techniques (21, 22). In this series, wound healing was the most frequent complication and was approach-related. We found that patients who underwent transoral/transmandibular procedures had a higher risk of perioperative complications. Steinberger et al. (2016) reviewed the safety of the transoral approach to the cervical spine in 126 patients (13). They found that this approach carries significant risks for morbidity (21.4%) and mortality (2.4%). In previous case reports on the transmandibular approach, the risk of approach-related complications was extremely high (8, 12, 33–35).

The retropharyngeal approach, also termed the high cervical or submandibular approach, is one of the safest and most effective methods to access the upper cervical spine as it provides wide exposure and feasibility for instrumentation, allowing for extension to the lower cervical spine (11). Yang et al. (2011) adopted a combined retropharyngeal-posterior approach in a cohort of 11 C2 tumors and found that one major and two minor approach-related complications occurred (2). Thus, we recommend the retropharyngeal approach for anterior procedures in most cases. However, this approach may not easily expose the C1 anterior arch and odontoid process in some cases. Endoscopic visualization may facilitate surgical manipulation (37).

### 4.3 Personalized AVBs: Better choice with minor pitfalls

3D-printed AVBs are ideal materials for large bone defects after the surgical resection of spinal tumors (10, 17–22). Biomechanical analysis revealed that the load of the head is transmitted *via* the bilateral C1/2 joints and then redistributes this two-column load into a three-column system of the subaxial spine (38). Thus, conventional or modified cylindrical titanium meshes do not comply with this biomechanical role well. Personalized titanium-alloy AVBs are fabricated with high fidelity to the structure of bony defects and can play an axial biomechanical role perfectly. Finite element analysis has shown that personalized AVB can increase the stability of the upper cervical segment and produce less stress on the C3 endplate than the Harms-modified mesh (39).

After a long history of AVB application in 2014 (16, 18, 20–22), we found that personalized AVB can superbly mimic the structures of resected tumor lesions and thus provide a reliable reconstruction of the column. At the same time, 3D-printed AVBs could provide immediate stability due to perfect structural conformability and spared the long-lasting use of the halo vest after the operation (**Table 1**). More importantly, the microstructure of AVBs mimics porous cancellous bone and heavily elevates osseointegration between the host bone and implants (40). In addition, the porous structure of AVBs facilitates the possibility of loading pharmaceuticals such as rhBMP-2, hydroxyapatite, antibiotics, and anti-tumor drugs (41, 42).

In this single-center comparative study, the risk of hardware problems in AVB patients was marginally lower than that in conventional titanium meshes. However, we noticed some minor pitfalls during our study. For example, we found it difficult to nail C1 screws in (case #B7) because of the block of the mandible. We believe that an embedded oblique screw trajectory may prevent this problem. Thus, the current design of personalized AVB requires additional modifications in the future.

### 4.4 Limitations

To begin with, the sample size of this study was small, and the results of the statistical analysis call for cautious interpretation. Additionally, considering its retrospective nature, this study does not provide evidence of high-level quality to address the superiority of personalized AVB but rather a case series. Furthermore, some patients received a short to medium follow-up period, while the results of this study require long-term examination.

## Data availability statement

The original contributions presented in the study are included in the article/**Supplementary Material**. Further inquiries can be directed to the corresponding author.

## Ethics statement

The studies involving human participants were reviewed and approved by Peking University Third Hospital Ethics Committee Board. The patients/participants provided their written informed consent to participate in this study.

## Author contributions

PH and SD designed the study, reviewed the patients, collected and processed the clinical data, and drafted the manuscript. FW designed the study, selected the patients, processed the data, and supervised the study. SZ and HZ reviewed the patients and collected the data. XL and ZL supervised the data collection and processing. All authors reviewed and approved this manuscript.
