# Supplementary material for: Reconstruction after resection of C2 vertebral tumors: A comparative study of 3D-printed vertebral body versus titanium mesh
Source: Front Oncol. 2022 Dec 19;12:1065303. doi: 10.3389/fonc.2022.1065303 (PMC9806260; doi:10.3389/fonc.2022.1065303)
Supplement: Supplementary file 1 [file Table_1.docx]

Supplementary table: Presentation of detailed demographic and clinical data

| Case No. | Sex/age (years) | Symptoms | Pathology | Staging | | Anterior approaches | Margin | Anterior reconstruction | Bleeding (posterior/anterior) | Perioperative events | Hardware problems | Halo-vest | Follow-ups (m) & survival |
| --- | --- | --- | --- | --- | --- | --- | --- | --- | --- | --- | --- | --- | --- |
|  |  |  |  | Enneking | WBB |  |  |  |  |  |  |  |  |
| Group A | | | | | | | | | | | | | |
| 1 | F/22 | neck pain | GCT | S3 | 2-11/A-D | transoral | I | titanium mesh | 800/400 | none | Malunion | Yes/7m | 144/Alive |
| 2 | M/34 | neck pain | GCT | S3 | 4-8/B-D | retropharyngeal | I | titanium mesh | 1250/1300 | poor wound healing | None | Yes/3m | 15/Alive |
| 3 | M/38 | neck pain | GCT | S3 | 3-10/B-C | transoral | I | titanium mesh | 2200/2000 | none | Disunion and move | Yes/2m | 105/Alive |
| 4 | M/28 | neck pain, UE numbness | osteoblastoma | S2 | 3-12/A-D | transoral | I | titanium mesh | 300/500 | PVAE, nerve sacrifice, CSF leakage, poor wound healing | None | Yes/3m | 12/Alive |
| 5 | M/58 | neck pain | osteoblastoma | S2 | 4-9/B-C | retropharyngeal | I | titanium mesh +LP | 1500/2500 | none | None | Yes/3m | 98/Alive |
| 6 | F/57 | neck pain | chordoma | IB | 2-9/A-D | transoral+transcervical | I | titanium mesh | 1000/1000 | VA and nerve sacrifice, poor wound healing | Failure | Yes/3m | 118/Alive |
| 7 | F/57 | neck pain | chordoma | IB | 5-10/A-C | transoral | I | titanium mesh +LP | 500/2000 | wound infection | None | Yes/3m | 96/Alive |
| 8 | M/26 | neck pain | chordoma | IB | 5-12/B-D | transmandibular | I | titanium mesh | 1000/3000 | VA and nerve sacrifice, cardiac event | None | No | 13/Alive |
| 9 | M/49 | torticollis | chordoma | IB | 2-11/A-D | transoral+ retropharyngeal | I | titanium mesh | 500/800 | VA sacrifice, poor wound healing, CSF leakage | Disunion and move | Yes/8m | 41/Alive |
| 10 | M/34 | UE and LE weakness | chordoma | IB | 3-10/A-D | transoral | I | titanium mesh +LP | 400/1200 | poor wound healing, CSF leakage | None | Yes/2m | 72/Dead |
| 11 | M/53 | neck pain, torticollis | chordoma | IB | 3-10/A-D | transoral | I | titanium mesh | 1200/2000 | nerve sacrifice, died (hemorrhagic shock) | None | / | / |
| 12 | M/51 | neck pain, UE numbness and weakness | hemangiopericytoma | IIB | 3-10/B-D | transoral | I | titanium mesh +LP | 500/800 | CSF leakage, wound infection | None | Yes/1m | 156/Dead |
| 13 | M/56 | UE and LE weakness | Schwannoma | S3 | 4-10/A-D | retropharyngeal | I | titanium mesh +LP | 500/500 | nerve sacrifice | None | No | 24/Alive |
| Group B | | | | | | | | | | | | | |
| 1 | F/16 | neck pain | GCT | S3 | 3-10/B-D | retropharyngeal | I | AVB | 400/300 | none | None | No | 62/Alive |
| 2 | F/24 | neck pain | GCT | S3 | 6-10/B-C | retropharyngeal | I | AVB | 900/200 | none | None | No | 88/Alive |
| 3 | M/40 | neck pain | GCT | S3 | 4-9/B-C | retropharyngeal | M | AVB | 600/1000 | none | None | No | 53/Alive |
| 4 | F/56 | neck pain | GCT | S3 | 3-10/B-C | transoral | I | AVB | 800/150 | none | None | No | 32/Alive |
| 5 | F/26 | neck pain | GCT | S3 | 3-10/B-C | retropharyngeal | I | AVB | 700/2000 | VA sacrifice | None | No | 20/Alive |
| 6 | F/18 | neck pain | GCT | S3 | 3-10/A-D | retropharyngeal | I | AVB | 300/200 | none | None | No | 27/Alive |
| 7 | M/20 | neck pain | GCT | S3 | 3-10/B-C | retropharyngeal | I | AVB | 200/100 | PVAE | Screw loose | Yes/3w | 14/Alive |
| 8 | F/31 | neck pain | GCT | S3 | 3-10/B-C | retropharyngeal | M | AVB | 1200/300 | none | None | No | 37/Alive |
| 9 | F/59 | neck pain | chordoma | IA | 4-9/B-D | retropharyngeal | M | AVB | 300/600 | none | None | No | 61/Alive |
| 10 | M/62 | neck pain | chordoma | IB | 2-10/A-D | retropharyngeal | I | AVB | 1000/300 | tracheotomy | None | No | 14/Alive |
| 11 | M/49 | neck pain | chordoma | IB | 3-10/B-C | retropharyngeal | I | AVB | 200/400 | CSF leakage, wound infection | None | Yes/8w | 35/Alive |
| 12 | F/47 | neck pain, UE and LE numbness | chordoma | IB | 6-11/A-D | retropharyngeal | I | AVB | 700/2000 | VA sacrifice, DVT/PE | None | Yes/6w | 44/Alive |
| 13 | M/58 | dysphagia | chordoma | IA | 4-9/A-C | retropharyngeal | I | AVB | 200/400 | poor wound healing, tracheotomy | None | No | 36/Alive |
| 14 | M/45 | neck pain | chordoma | IB | 4-9/A-D | retropharyngeal | I | AVB | 750/500 | none | None | No | 14/Alive |
| 15 | F/46 | neck pain | chordoma | IB | 1-12/A-D | retropharyngeal | I | AVB | 1300/400 | none | None | No | 44/Alive |
| 16 | F/31 | dyspnea | chordoma | IB | 7-11/A-D | retropharyngeal | I | AVB | 800/600 | died (respiratory failure, septic shock), tracheotomy | None | / | / |
| 17 | F/47 | neck pain, UE and LE weakness | paraganglioma | IIB | 4-9/B-D | retropharyngeal | I | AVB | 500/800 | none | None | No | 13/Alive |
| 18 | M/12 | neck pain | Ewing sarcoma | IIB | 1-12/B-D | retropharyngeal | I | AVB | 400/600 | none | None | No | 15/Dead |

UE stands for upper extremity; LE, lower extremity; GCT, giant cell tumor; LP, locking plate; AVB, artificial vertebral body; VA, vertebral artery; PVAE, preoperative vertebral artery embolization; DVT, deep venous thrombosis ;PE, pulmonary embolism.
